# Identification of a *Chlamydomonas* plastidial 2‐lysophosphatidic acid acyltransferase and its use to engineer microalgae with increased oil content

**DOI:** 10.1111/pbi.12572

**Published:** 2016-05-23

**Authors:** Yasuyo Yamaoka, Dorine Achard, Sunghoon Jang, Bertrand Legéret, Shogo Kamisuki, Donghwi Ko, Miriam Schulz‐Raffelt, Yeongho Kim, Won‐Yong Song, Ikuo Nishida, Yonghua Li‐Beisson, Youngsook Lee

**Affiliations:** ^1^ Department of Life SciencePohang University of Science and TechnologyPohangKorea; ^2^ Institut de Biosciences et BiotechnologiesCEA CadaracheSaint‐Paul‐lez‐DuranceFrance; ^3^ Division of Life ScienceGraduate School of Science and EngineeringSaitama UniversitySakura‐KuSaitamaJapan; ^4^ JSTCRESTChiyoda‐kuTokyoJapan; ^5^ Department of Integrative Bioscience & BiotechnologyPohang University of Science and TechnologyPohangKorea; ^6^ Present address: School of Biological Sciences and Center for Plant Science InnovationUniversity of Nebraska LincolnLincolnNEUSA

**Keywords:** microalgae, lysophosphatidic acid acyltransferase, triacylglycerols, plastid transformation, acyl specificity, oil content

## Abstract

Despite a strong interest in microalgal oil production, our understanding of the biosynthetic pathways that produce algal lipids and the genes involved in the biosynthetic processes remains incomplete. Here, we report that *Chlamydomonas reinhardtii Cre09.g398289* encodes a plastid‐targeted 2‐lysophosphatidic acid acyltransferase (CrLPAAT1) that acylates the *sn*‐2 position of a 2‐lysophosphatidic acid to form phosphatidic acid, the first common precursor of membrane and storage lipids. *In vitro* enzyme assays showed that CrLPAAT1 prefers 16:0‐CoA to 18:1‐CoA as an acyl donor. Fluorescent protein‐tagged CrLPAAT1 was localized to the plastid membrane in *C. reinhardtii* cells. Furthermore, expression of CrLPAAT1 in plastids led to a > 20% increase in oil content under nitrogen‐deficient conditions. Taken together, these results demonstrate that CrLPAAT1 is an authentic plastid‐targeted LPAAT in *C. reinhardtii*, and that it may be used as a molecular tool to genetically increase oil content in microalgae.

## Introduction

Glycerolipids are one of the most abundant lipid classes in nature, and exist in both prokaryotes and eukaryotes. The assembly of glycerolipids starts with stepwise acylation of glycerol 3‐phosphate (G3P) to phosphatidic acid (PA) using fatty acyl donors. Glycerol‐3‐phosphate acyltransferase (GPAT; EC2.3.1.15) and 2‐lysophosphatidic acid acyltransferase (LPAAT, EC2.3.1.51) catalyse the acylation at the *sn*‐1 position of G3P and the *sn*‐2 position of 2‐lysophosphatidic acid (LPA), to produce LPA and PA, respectively. These enzymes have been well studied in the higher model plant *Arabidopsis thaliana*, where two sets of homologous enzymes catalyse two distinct and parallel glycerolipid biosynthesis pathways, one in the plastid (the ‘prokaryotic pathway’) and the other in the endoplasmic reticulum (ER, the ‘eukaryotic pathway’) (Ohlrogge and Browse, 1995; Roughan and Slack, 1982). Plants can be divided into two major types, that is 16 : 3 or 18 : 3, depending on the origin of their plastidial lipids (Roughan and Slack, 1982). Glycerolipids of prokaryotic origin contain 16 : 3 fatty acids at the *sn*‐2 position, whereas those of eukaryotic origin contain 18 : 3 fatty acids. A survey of over 400 plant species across different taxa suggests that the plastidial pathway was lost during the evolution from a single‐celled cyanobacterium to some higher vascular plants, such as *Glycine max* (soybean; Mongrand *et al*., 1998). By contrast, some plant species, including *Arabidopsis thaliana,* still employ both pathways for the biosynthesis of thylakoid lipids. The contribution of each pathway can vary and is species specific. The products of the two pathways are generally distinguished based on their fatty acid species at the *sn*‐2 position, which are determined by the acyl specificity of the LPAAT homologs in the two pathways (Frentzen, 1993; Heinz and Roughan, 1983). Studies of LPAATs can thus provide clues into the fluxes of lipid metabolites.

In most characterized species, the plastidic LPAAT has a strong preference for C16 fatty acids, while the ER‐localized LPAAT has a high specificity for C18 fatty acids (Kim and Huang, 2004; Kim *et al*., 2005; Yu *et al*., 2004). The thylakoid membrane lipids from the unicellular green microalga *Chlamydomonas reinhardtii* retain only the prokaryotic configuration at the *sn*‐2 positions (Giroud *et al*., 1988). Increasing evidence suggests that the prokaryotic pathway also plays an important role in the biosynthesis of storage lipids, that is triacylglycerols (TAGs), in this alga (Fan *et al*., 2011; Goodson *et al*., 2011). Despite the central position that LPAAT occupies in the biosynthesis of both membrane and storage lipids, no putative LPAATs encoded in the *C. reinhardtii* genome have been characterized to date. Thus, it is not clear how many functional LPAATs exist in *Chlamydomonas*, or whether *Chlamydomonas* LPAATs possess the same acyl specificities as those of higher plants. These issues are important not only for understanding the subcellular organization of lipid metabolic pathways, but they also have implications for our understanding of lipid transport processes and for our ability to genetically manipulate microalgae to have increased oil content.

Here, we characterize a plastid‐targeted LPAAT from *C. reinhardtii*, which is putatively annotated under the genetic code of *Cre09.g398289*. We show that the recombinant protein encoded by *Cre09.g398289* exhibits LPAAT activity with a preference for C16 over C18 fatty acyl substrates. We also demonstrate that CrLPAAT1 is targeted to plastid membranes in *Chlamydomonas* and tobacco cells, and that its overexpression boosts oil content in *C. reinhardtii*. We discuss the implications of these findings in the context of the subcellular organization of lipid metabolism in *C. reinhardtii*.

## Results

### *Chlamydomonas* has one putative plastidial lysophosphatidic acid acyltransferase, CrLPAAT1

**Figure 1:**
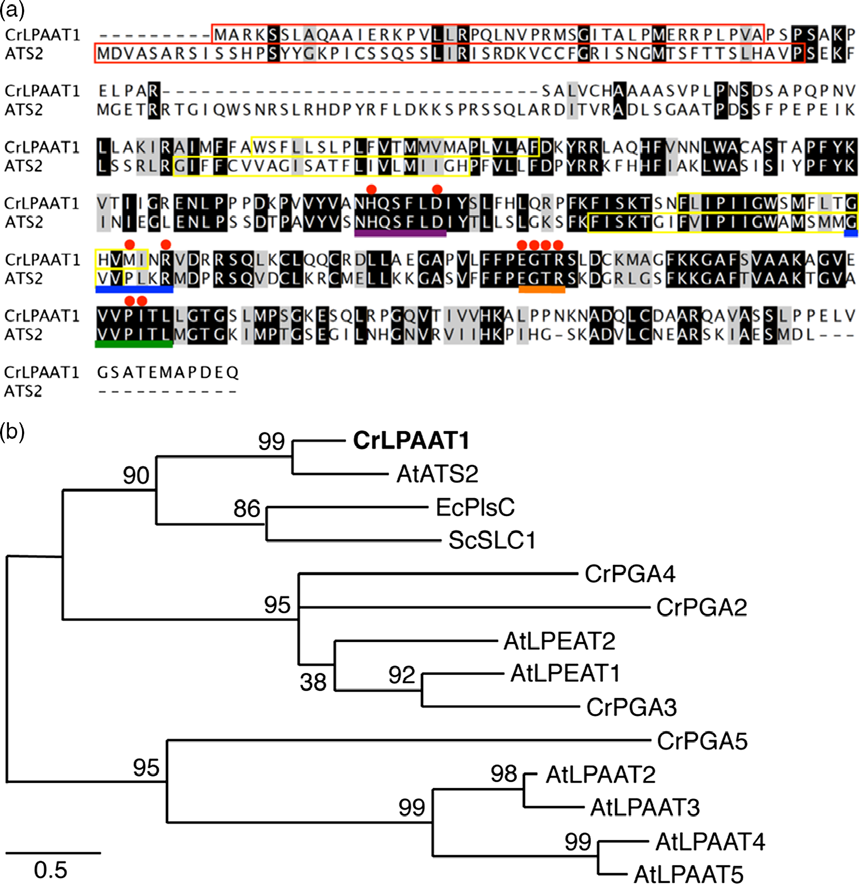
CrLPAAT1 is a homolog of the Arabidopsis plastidial protein LPAAT1 (ATS2). (a) Amino acid sequence alignment of CrLPAAT1 and AtATS2/AtLPAAT1. Black and grey boxes represent conserved and similar residues, respectively. Red boxes in the first row of the alignment indicate the plastid targeting transit peptides predicted using PredAlgo (Tardif *et al*., 2012). Motifs and amino acids identified as being important (Yamashita *et al*., 2007) are highlighted: NHX4D (motif I, purple line), GVIFIDR (motif II, blue line), EGTR (motif III, orange line), IVPIVM (motif IV, green line) and amino acid residues (red dots). Yellow boxes indicate hypothetical membrane‐spanning domains predicted by TMHMM (Krogh *et al*., 2001). (b) A phylogenetic tree of lysophosphatidic acid acyltransferase based mostly on sequences from *C. reinhardtii* and *Arabidopsis thaliana*. The protein sequences of *C. reinhardtii* were obtained from Phytozome v10.3: CrLPAAT1 (Cre09.g398289.t1.1); CrPGA2 (Cre05.g248150.t1.2); CrPGA3 (Cre17.g707300.t1.2); CrPGA4 (Cre10.g460350.t1.1); and CrPGA5 (Cre17.g738350.t1.2). The protein sequences of *A. thaliana* were obtained from TAIR: AtATS2/AtLPAAT1 (At4 g30580.1); AtLPAAT2 (At3 g57650.1); AtLPAAT3 (At1 g51260.1); AtLPAAT4 (At1 g75020.1); AtLPEAT1 (At1 g80950.1); and AtLPEAT2 (At2 g45670.1). LPAATs of *Escherichia coli* (EcPlsC, M63491) and *Saccharomyces cerevisiae* (ScSLC1, L13282) are included. The phylogenetic tree was inferred using MEGA6 and the maximum‐likelihood method with 1000 bootstrap replications. LPAAT: Lysophosphatidic acid acyltransferase, PGA: Phospholipid/Glycerol Acyltransferase. The percentage of trees in which the associated taxa clustered together is shown next to the branches. Scale bar represents substitutions per unit distance.

To identify an ortholog of *Chlamydomonas* plastid‐targeted LPAAT, the *Chlamydomonas* genome database at Phytozome V10.3 was searched for homologs of Arabidopsis ATS2 (AtLPAAT1: At4 g30580) that encode plastidial LPAAT (Yu *et al*., 2004). The protein encoded by the locus Cre09.g398289 showed the highest level of amino acid sequence identity (i.e. 40%) to AtATS2 (Figure 1a). Expressed sequence tags (ESTs) for *Cre09.g398289* were found in Phytozome V10.3, suggesting that this locus is expressed in *Chlamydomonas* cells. We designated this protein CrLPAAT1. CrLPAAT1 contains canonical motifs attributed to LPAAT proteins (Kim *et al*., 2005), such as NHX4D (motif I), GVIFIDR (motif II), EGTR (motif III) and IVPIVM (motif IV) (highlighted in Figure 1a). CrLPAAT1 also contains the hypothetical membrane‐spanning domains, as predicted by transmembrane helix prediction (TMHMM, Krogh *et al*., 2001) (yellow boxes in Figure 1a). CrLPAAT1 has a putative plastid transit peptide (~51 amino acids; enclosed in a red box in Figure 1a**)** as predicted by PredAlgo (Tardif *et al*., 2012), suggesting that CrLPAAT1 could be targeted to the plastid in *C. reinhardtii*.

In addition to CrLPAAT1, six proteins have been annotated in the Phytozome *C. reinhardtii* database as phospholipid/glycerol acyltransferases 1–6 (PGA1–PGA6) (Table 1; Figure 1b) (Merchant *et al*., 2007). PGA1 and PGA6 belong to the Tafazzin (TAZ) protein family, and are likely involved in cardiolipin transacylation in the mitochondrial phospholipid biosynthesis pathway, and are therefore important for the remodelling of acyl groups in cardiolipin (Xu *et al*., 2006). PGA2, PGA3 and PGA4 showed high levels of sequence identity to the two known Arabidopsis lysophosphatidylethanolamine acyltransferases (LPEATs), that is AtLPEAT1 (At1 g80950; 37.2%, 31.0% and 28.3%, respectively) and AtLPEAT2 (At2 g45670; 21.9%, 23.5% and 20.3%, respectively). Biochemical analysis of the two Arabidopsis LPEAT proteins heterologously expressed in yeast showed that both proteins preferentially use lysophosphatidylethanolamine (LPE) as a substrate to form PtdEtn (Stalberg *et al*., 2009), but no evidence is available for their *in planta* function. None of the PGA proteins is predicted to be plastidial, and all of them (PGA1–6) are putatively targeted to the secretory pathway (based on PredAlgo prediction, Table 1). No experimental evidence is available regarding their function *in vivo*.

**Table 1:** Putative proteins involved in converting glycerol 3‐phosphate (G3P) to lysophosphatidic acid (LPA) and phosphatidic acid (PA)

Enzyme family	JGI v5.5 ID	Gene name abbreviation	Description	Localization	Homologs in Arabidopsis
GPAT	Cre02.g143000	CrGPA1	Glycerol‐3‐phosphate O‐acyltransferase	C	At1 g32200 (ATS1/ACT1)
	Cre06.g273250	CrGPA2	Glycerol‐3‐phosphate O‐acyltransferase	C	At5 g60620 (GPAT9)
LPAAT	Cre09.g398289	CrLPAAT1	1‐acyl‐sn‐glycerol‐3‐phosphate acyltransferase	C	At4 g30580 (LPAAT1/ATS2)
	Cre17.g707300	CrPGA3	Phospholipid/glycerol acyltransferases	SP	At1 g80950 (LPEAT1)
	Cre05.g248150	CrPGA2	Phospholipid/glycerol acyltransferases	SP	At2 g45670 (LPEAT2)
	Cre10.g460350	CrPGA4	Phospholipid/glycerol acyltransferases	SP	At1 g80950 (LPEAT1)
	Cre17.g738350	CrPGA5	Phospholipid/glycerol acyltransferases	SP	–
	Cre11.g467850	CrPGA6	Phospholipid/glycerol acyltransferases	SP	At3 g05510 (TAZ1)
	Cre03.g182650	CrPGA1	Phospholipid/glycerol acyltransferases	SP	At3 g05510 (TAZ1)

Protein subcellular localization was predicted using PredAlgo (https://giavap-genomes.ibpc.fr/cgi-bin/predalgodb.perl?page=main) (Tardif *et al*., 2012). C, chloroplast; SP, secretory pathway.

### CrLPAAT1 functionally complements the high temperature sensitivity of the *Escherichia coli plsC* mutant, which is deficient in LPAAT

**Figure 2:**
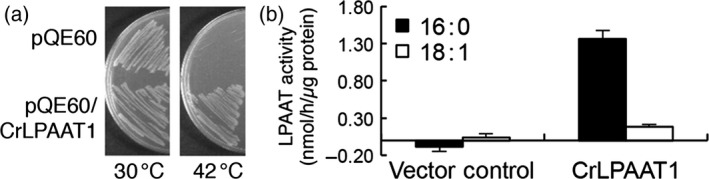
CrLPAAT1 functions as an LPAAT. (a) CrLPAAT1 expression rescued the high temperature sensitivity of the *E. coli plsC* mutant, which is deficient in LPAAT. (b) Membrane fractions (60 μg protein) isolated from the transgenic *E. coli* cells catalysed phosphatidic acid formation in the presence of 200 μm LysoPA‐18:1, 25 μm 18:1‐CoA (white bars) and 25 μm 16:0‐CoA (black bars). Data are means of three replicates with standard deviations shown. Note that the buffer control has been subtracted from individual results.

The *E. coli* strain SM2‐1, which harbours a mutation in the LPAAT gene *plsC*, grows well at 37 °C, but cannot grow at an elevated temperature (i.e. 42 °C) (Coleman, 1990). This strain has been extensively used for functional studies of LPAAT genes from various organisms (Kim and Huang, 2004). We next conducted a functional analysis of *Chlamydomonas* CrLPAAT1 using this strain. Due to the high GC content of the native *Chlamydomonas* gene (64% GC) (Merchant *et al*., 2007), we first optimized the codon usage of CrLPAAT1 to be expressed in *E. coli* cells. *E. coli* SM2‐1 cells expressing the codon‐optimized gene (*CrLPAAT1e*) grew well at 42 °C, but those containing the empty vector (pQE60) did not grow under the same conditions (Figure 2a). These results suggest that CrLPAAT functions as a LPAAT in *E. coli* cells.

### CrLPAAT1 prefers 16:0‐CoA over 18:1‐CoA as an *in vitro* substrate

We then examined the substrate selectivity of CrLPAAT1, using membrane fractions of *E. coli plsC* cells expressing *CrLPAAT1e*. As shown in Figure 2b, the membrane fraction from *E. coli* expressing *CrLPAAT1e* exhibited LPAAT activity, with 7.6‐fold higher activity with 16:0‐CoA than with 18:1‐CoA. The membrane fractions from vector control cells did not produce PA (Figure 2b). These results demonstrate that CrLPAAT1 is a functional LPAAT that prefers C16 over C18 substrate.

### CrLPAAT1 is targeted to the plastid

**Figure 3:**
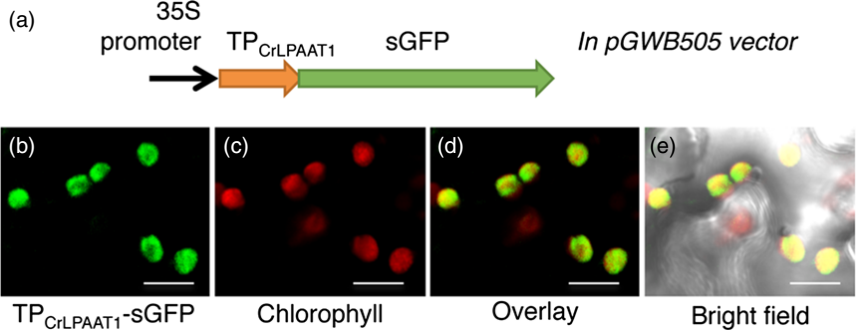
Green fluorescent protein (GFP) was targeted to the plastid when fused to the transit peptide (TP) of CrLPAAT1 protein in tobacco epidermal cells. (a) A schematic diagram of the construct used to test the putative transit peptide of CrLPAAT1. (b) GFP fluorescence when tobacco epidermal cells were transformed with the TP_C__r_ _LPAAT__1_‐sGFP fusion protein. (c) Chlorophyll autofluorescence. (d) Overlay of TP_C__r_ _LPAAT__1_‐sGFP and chlorophyll fluorescence. (e) Overlay of TP_C__r_ _LPAAT__1_‐sGFP, chlorophyll fluorescence and bright field. The construct was agro‐infiltrated into tobacco leaves and visualized by confocal microscopy. Bars = 10 μm.

The preferential acylation of the *sn*‐2 position of LPA with 16:0‐CoA over 18:1‐CoA suggests that CrLPAAT1 is a plastidic isoform of LPAAT. To test whether CrLPAAT1 is indeed targeted to plastids, we fused an *N*‐terminal fragment (86 amino acids) of CrLPAAT1 to sGFP, and transiently expressed the resultant construct in tobacco leaves under the control of the cauliflower mosaic virus 35S promoter, using an *Agrobacterium*‐mediated infiltration method. Confocal laser scanning microscopy showed that sGFP fluorescence is associated with plastids in tobacco leaf epidermal cells (Figure 3), suggesting that the 86‐amino acid *N*‐terminal sequence of CrLPAAT1 functions as a transit peptide for targeting to plastids in tobacco cells and, hence, most likely in *Chlamydomonas* cells, too.

**Figure 4:**
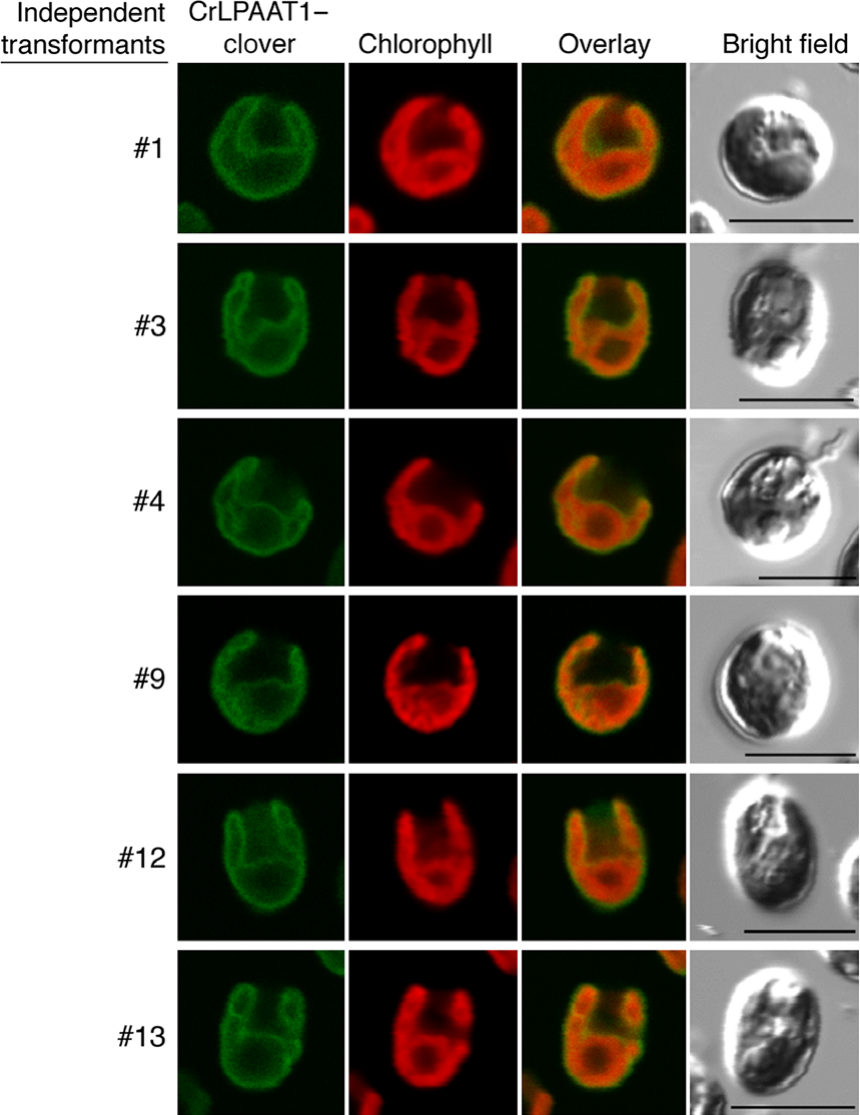
CrLPAAT1 was localized to *C. reinhardtii* plastids. *Chlamydomonas reinhardtii* (strain CC‐125) was transformed with pOpt‐CrLPAAT1‐Clover. From left to right: Clover fluorescence, chlorophyll fluorescence, overlay of Clover and chlorophyll fluorescence, and bright field. The numbers to the left of each figure indicate the name of each individual transformant expressing *CrLPAAT1–Clover*. Bars = 10 μm.

To further examine the subcellular localization of a full‐length construct of a fluorescent protein‐tagged CrLPAAT1 in *C. reinhardtii* (Figure 4), we fused the fluorescence protein Clover to the C‐terminus of CrLPAAT1. We expressed the resultant CrLPAAT1–Clover fusion construct in *C. reinhardtii* cells under the control of a chimeric promoter consisting of a fusion of the heat‐shock protein 70A (HSP70A) promoter and the Rubisco small subunit 2 (RBCS2) promoter (Schroda *et al*., 2000). Figure 4 shows confocal laser scanning microscopy images of six representative independent transformant lines. Clover fluorescence was largely confined to the cup‐shaped plastid envelope (see yellow‐orange fluorescence in the overlay panel). This result is consistent with that of the *in planta* transient expression assay, and indicates that CrLPAAT1 is a plastidial isoform of LPAAT in *C. reinhardtii*. This observation serves as an example of a chloroplast transit peptide that can function in both higher plants and *Chlamydomonas* (Figure 3).

### *CrLPAAT1* expression is induced by nitrogen starvation

**Figure 5:**
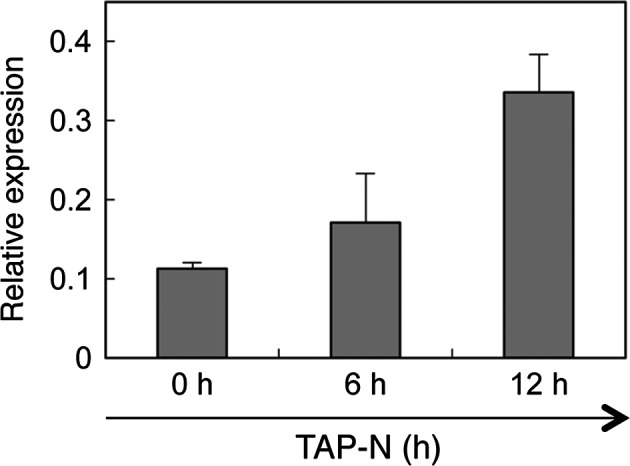
Quantitative RT‐PCR analysis of the transcript level of *CrLPAAT1* under nitrogen starvation. Expression levels of *CrLPAAT1* were normalized to that of the housekeeping gene *RACK1*. Error bars represent standard errors based on three biological replicates.

Real‐time quantitative RT‐PCR analysis showed that moderate levels of *CrLPAAT1* transcript were detected in mid‐log phase cells grown at 25 °C (0 h), whereas levels had increased threefold by 12 h after transfer to TAP medium lacking nitrogen (TAP–N medium) (Figure 5). These data suggest that *CrLPAAT1*, like many other lipid biosynthesis genes (Boyle *et al*., 2012; Miller *et al*., 2010), is up‐regulated in response to nitrogen starvation. This increase in *LPAAT1* transcript suggests its possible role in nitrogen starvation‐induced TAG accumulation, as reported below.

### Overexpression of *CrLPAAT1* in *C. reinhardtii* cells by plastid genome transformation increases oil content

**Figure 6:**
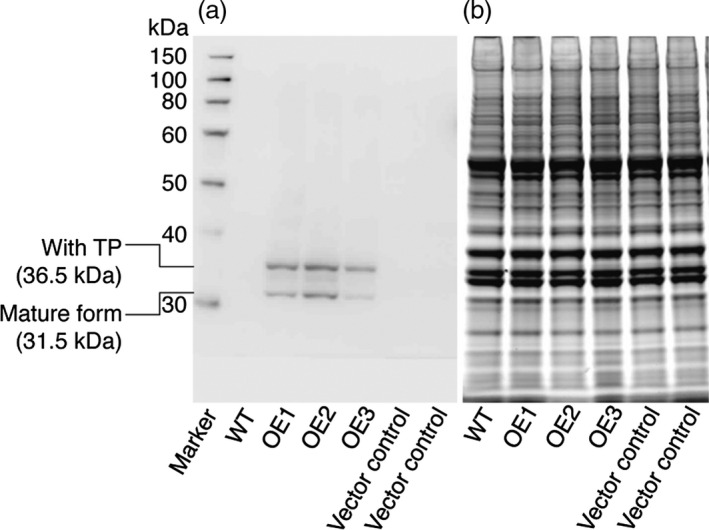
Immunodetection of CrLPAAT1 expression in *C. reinhardtii* cells expressing HA‐tagged CrLPAAT1 using anti‐HA antibodies. (a) Immunoblot analysis of *C. reinhardtii* cells expressing HA‐tagged CrLPAAT1 using anti‐HA antibodies. Two bands were detected; the upper band corresponds to the full protein (36.5 kDa), and the lower band corresponds to the mature protein (31.5 kDa) lacking the transit peptide. (b) The SDS‐PAGE gel was stained with blue dye (ProSieve EX Safe Stain—LONZA) as a loading control for the immunoblot shown in (a). Twenty micrograms of total proteins were loaded onto an SDS‐PAGE gel, and expression of CrLPAAT1 was detected using anti‐HA antibodies. A duplicate of the gel was also visualized after staining with blue dye for 1 h. OE: pLM21‐CrLPAAT1‐HA overexpressors; three transformants (OE1, OE2 and OE3) are shown.

Several recent studies have revealed that the ‘prokaryotic’ diacylglycerol moieties contribute extensively to TAG synthesis in *C. reinhardtii* cells under nutrient starvation and other stress conditions (Fan *et al*., 2011; Goodson *et al*., 2011). Here, we tested the impact of *CrLPAAT1* on oil accumulation by overexpressing CrLPAAT1 (CrLPAAT1‐HA) in the *C. reinhardtii* plastid genome. To this end, the codon‐optimized gene *CrLPAAT1‐HA* was synthesized as described in materials and methods and inserted together with a spectinomycin resistance marker gene into the plastid genome by homologous recombination. The resultant spectinomycin‐resistant clones were successively selected for spectinomycin resistance with increasing spectinomycin concentrations from 100 μg/mL to 300 μg/mL, a practice commonly used to bring cells to the homoplasmic state (Figure S1). Three independent homoplasmic clones (OE1–OE3) were verified for CrLPAAT1‐HA overexpression by immunoblotting using anti‐HA antibodies (Figure 6). Interestingly, two forms of the protein were detected on the immunoblot; the upper band (~36 kDa) corresponds to the preprotein still containing the plastid transit peptide (TP ≈ 5 kDa), whereas the lower band corresponds to the mature protein (~31 kDa) without the transit peptide.

**Figure 7:**
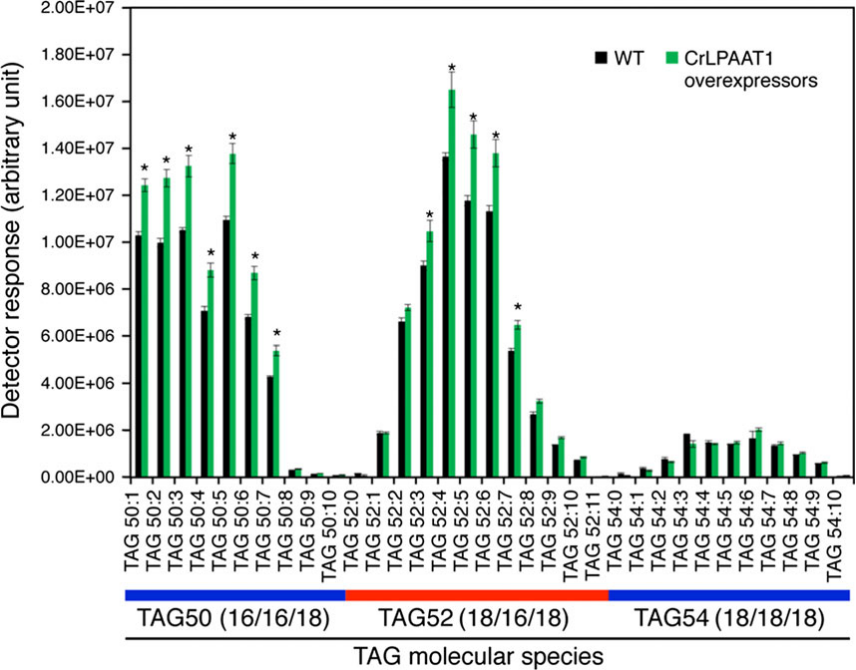
Oil content is increased by up to 20% in plastidial transgenic lines overexpressing CrLPAAT1 under nitrogen starvation. Cells were harvested from nitrogen‐starved cells (TAP‐N for 3 days). Data are means of three biological replicates (OE1, OE2 and OE3 shown in Figure 6) together with three technical replicates for each biological replicate; error bars denote 95% confidence intervals. *: denotes significant increases. Statistical analysis was carried out using the Student's *t*‐test (*P *<* *0.05).

The CrLPAAT1‐HA overexpressing lines were tested for TAG accumulation under nitrogen starvation. Total lipids were extracted and subjected to LC‐MS/MS analysis. OE lines showed a substantial increase (~20%) in TAG content under nitrogen starvation (Figure 7). As expected from the substrate specificity of CrLPAAT1, this increase was represented by the TAG molecular species TAG50 and TAG52, both of which contained C16 fatty acids, whereas the level of TAG54 molecular species, which contain only C18 fatty acids, did not change. Interestingly, polar membrane lipid levels also increased in our overexpressors, as shown in both Figure S2 and S3 for membrane lipid quantification during and before nitrogen starvation. These results indicate that CrLPAAT1 contributes to the biosynthesis of PA precursors to both storage and membrane lipids in *Chlamydomonas* plastids.

## Discussion

With the increasing interest in microalgae as a feedstock for bioenergy production, the lipid biosynthetic pathways in the green model microalga *C. reinhardtii* have been subjected to intensive studies over the past 10 years. Nevertheless, there are still many gaps in our understanding of the subcellular organization of TAG formation in this organism. For example, recent research demonstrated that besides the classical ER‐pathway, TAGs can also be assembled in *C. reinhardtii* plastids. This conclusion is largely based on transmission electron microscopy analysis, which revealed lipid droplets in plastids, and also on the chemical structures of newly formed TAGs, >90% of which contain a C16 fatty acid esterified at the *sn*‐2 position, which can only arise from DAG backbones made in the plastid (Fan *et al*., 2011; Goodson *et al*., 2011).

**Figure 8:**
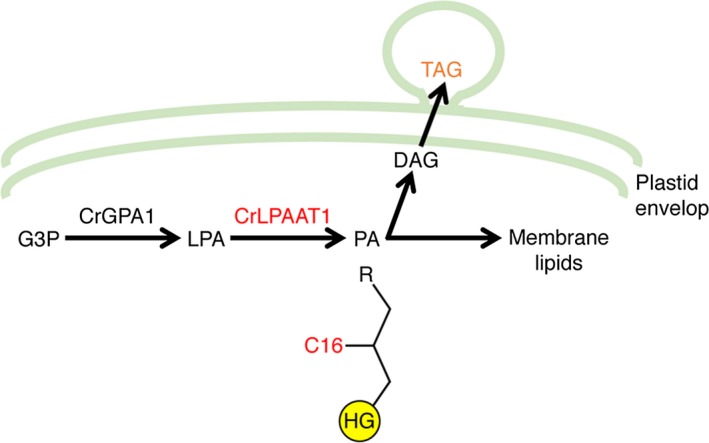
The involvement of CrLPAAT1 in oil synthesis in *C. reinhardtii*. HG, head group; G3P, glycerol‐3‐phosphate; LPA, lysophosphatidic acid; PA, phosphatidic acid; DAG, diacylglycerol; TAG, triacylglycerol; R, an acyl group. Arrows indicate catalytic steps, and enzymes are indicated above the arrows (CrLPAAT1 and C16 are in red).

Most active research has focused on the terminal acyltransferase that acylates DAGs to form TAGs (Boyle *et al*., 2012; La Russa *et al*., 2012; Yoon *et al*., 2012), and none of the LPAAT proteins from *C. reinhardtii* had hitherto been characterized. In this study, we characterized an LPAAT protein from *C. reinhardtii* in detail. We provided biochemical, genetic and subcellular localization data to demonstrate that Cre09.g398289 of *C. reinhardtii* encodes a functional plastid‐targeted LPAAT and prefers C16 fatty acids as a substrate. We further showed that its overexpression in the plastid resulted in a 20% increase in oil content, thus supporting previous observations (Fan *et al*., 2011; Goodson *et al*., 2011) that the plastidial pathway contributes to oil synthesis in *C. reinhardtii* (Figure 8). In addition, polar membrane lipid levels also increased in our overexpressors (Figures S2 and S3), suggesting that this plastidial LPAAT is important for both neutral and membrane lipid synthesis.

The function of plastidial LPAATs seems to be widely conserved from bacteria to microalgae and higher eukaryotes, as LPAATs of higher plants and *Chlamydomonas* can complement an *E. coli* mutant deficient in LPAAT. A recent study on the evolution of LPAATs (Korbes *et al*., 2015) confirmed this notion that the function of plastidial LPAATs is widely conserved. Similar to *C. reinhardtii*, other microalgae with sequenced genomes, including *Volvox carteri, Ostreococcus lucimarinus* and *Coccomyxa subellipsoidea,* possess a single putative plastidial LPAAT that belongs to the same subcluster as LPAAT1s from plants and cyanobacteria, suggesting a common origin of the plastidic isoform of LPAAT1.

However, the situation is different for ER‐localized LPAATs. We did not find any sequence in *C. reinhardtii* that showed homology to the four ER‐targeted Arabidopsis LPAAT proteins, that is AtLPAAT2–5 (Kim and Huang, 2004; Kim *et al*., 2005) (Figure 1b). This raises the question of how fatty acids at the *sn*‐2 positions of ER‐localized lipids, such as diacylglycerol *N,N,N*‐trimethylhomoserine (DGTS), PtdEtn and phosphatidylinositol (PtdIns), are acylated in *C. reinhardtii*. PGA2‐4 has been found to be associated with oil bodies in *C. reinhardtii* (Moellering and Benning, 2010; Nguyen *et al*., 2011), suggesting their likely involvement in oil synthesis. It remains to be determined whether the PGA proteins (PGA2‐5) can function as an endosomal plant‐type LPAAT. Establishing the identity and acyl specificity of the endosomal‐type LPAAT in *C. reinhardtii* will not only provide insight into how glycerolipids are assembled in this organism, but will also indicate whether or not an ER‐to‐plastid retrograde lipid import pathway exists (Li *et al*., 2015; Warakanont *et al*., 2015).

In addition to the above basic scientific findings, we provided an example of genetic engineering of plastid metabolism for augmentation of oil content; overexpression of CrLPAAT1 in the plastids by plastid genome transformation enhances the accumulation of TAG molecular species consisting of prokaryotic diacylglycerol moieties. A 16% increase in oil content via overexpression of endosomal LPAAT proteins has been reported in the seeds of the higher plant *Arabidopsis thaliana* (Maisonneuve *et al*., 2010). Nevertheless, to date, plastid genome transformation has only been used to produce wax esters (Aslan *et al*., 2014), biodegradable plastics (Poirier *et al*., 1995), carotenoids (Hasunuma *et al*., 2008) and recombinant pharmaceutical proteins (Bock, 2015). Our results establish that the plastid genome can be manipulated to increase oil production in algal cells, and could possibly be extended to vegetative cells in general. Oil production in vegetative tissues, such as leaves, in either the plastid or the cytosol, has the potential to yield much more oil from the same area than does production in a natural sink tissue such as seed or fruit (Ohlrogge *et al*., 2009). Thus, this study opens a new avenue of oil production that is based on genetically engineering plastid‐containing organisms (e.g. algae and plants).

## Experimental procedures

### Strains and culture conditions

The *Chlamydomonas* wild‐type strain CC‐125 (137C, *mt* ^*+*^) was obtained from the Chlamydomonas Genetics Center (USA). *Chlamydomonas* cells were cultured in a 250‐mL Erlenmeyer flask containing 100 mL Tris–acetate–phosphate (TAP) medium (Harris, 2001) at 25 °C under continuous light (75 μmol photons/m^2^/s) while shaking. Cell growth was monitored by measuring the optical density at 750 nm (OD_750_) with an Infinite M200 Pro plate reader (Tecan, Switzerland).

### DNA database searches and protein sequence analyses

The *C. reinhardtii* genome was searched using the BlastP program implemented in Phytozome v10.3 (http://phytozome.jgi.doe.gov/pz/portal.html) (Merchant *et al*., 2007) for proteins that show homology to plant, yeast and bacterial LPAATs. Protein sequence alignments were constructed using Clustal Omega (Sievers *et al*., 2011). Phylogenetic trees were inferred using MEGA6 and the maximum‐likelihood method with 1000 bootstrap replications (Tamura *et al*., 2013). The subcellular localization of each LPAAT candidate was predicted using the PredAlgo program (https://giavap-genomes.ibpc.fr/cgi-bin/predalgodb.perl?page=main) (Tardif *et al*., 2012).

### Functional complementation of an *Escherichia coli* mutant deficient in LPAAT (plsC) by expression of a codon‐optimized *CrLPAAT1* gene

As the *C. reinhardtii* genome has a relatively high GC content (Merchant *et al*., 2007), the codon usage of *CrLPAAT1* cDNA was optimized for expression in *E. coli* cells and, to facilitate cloning, additional *Nco*I and *BamH*I sites were inserted before the start codon and after the stop codon, respectively. This DNA sequence was then synthesized by Bioneer (Daejeon, Korea). The resultant codon‐optimized *CrLPAAT1* (designated *CrLPAAT1e*) was inserted as an *Nco*I–*BamH*I fragment into the bacterial expression vector pQE60 (Qiagen, Hilden, Germany). To test whether *CrLPAAT1e* could functionally complement the *E. coli plsC* mutation (Coleman, 1990), SM2‐1 cells, which harbour the *plsC* mutation, were transformed with pQE60‐*CrLPAAT1e* or pQE60 (vector control), and grown on LB agar plates at 30 °C or 42 °C (to evaluate their sensitivity to higher temperature).

### Measurement of LPAAT activity using recombinant CrLPAAT1

*Escherichia coli* SM2‐1 cells transformed with pQE60‐*CrLPAAT1e* and pREP4 were cultured in LB medium at 37 °C until the cultures reached an optical density (OD) at 600 nm (OD_600_) of 0.4. To induce protein expression, IPTG was added to the cell culture to a final concentration of 1.5 mm and the cells were further incubated at 23 °C for 18 h. Membrane fractions were prepared following the method described in Kim and Huang (2004) with slight modifications. Briefly, *E. coli* cells were resuspended in 4 mL resuspending medium containing 50 mm Tris–HCl (pH 8.0), 2 mm MgCl_2_ and 2 mm dithiothreitol (DTT), and disrupted by sonication with an ultrasonic generator (VC 130PB; SONICS, Newtown, CT). The total cell lysates were centrifuged at 10 000 ***g*** for 15 min at 4 °C. The supernatant was centrifuged at 100 000 ***g*** for 1.5 h at 4 °C. The membrane fraction was prepared by resuspending the pellet in 1 mL of resuspending medium, and LPAAT activity was measured in the resulting suspension.

LPAAT activity was measured as follows: the bacterial membrane fraction (equivalent to 60 μg protein) was added to reaction mixture (1000 μL in total) containing 50 mm Tris–HCl (pH 8.0), 1 mm MgCl_2_, 1 mm DTT, 200 μm 1‐oleoyl‐LPA (Avanti, Alabaster, AL) and 20 μm acyl‐CoA (18 : 1 and 16 : 0, Avanti). The mixture was incubated for 10 min at 30 °C (Kim and Huang, 2004). The reaction was terminated by adding chloroform:methanol (1 : 1 by volume). Lipids in the organic phase were applied to a thin‐layer chromatography (TLC) plate (Merck, Cat. No. 105721), and separated using a solvent mixture of acetone: toluene: water = 91 : 30 : 8, by volume. The spots of PA, which were identified by spraying the plate with 0.01% (w/v) primuline reagent (Sigma‐Aldrich Korea, Korea), were converted to methyl esters and then subjected to fatty acid quantification using gas chromatography–mass spectrometry (GC‐MS) as described in Kim *et al*. (2013).

### Subcellular targeting ability of the putative transit peptide of CrLPAAT1 using a transient expression system in tobacco leaf

The putative transit peptide sequence of CrLPAAT1 was predicted using PredAlgo, and the DNA sequence encoding the predicted N‐terminal 86‐amino acid residues (designated TP_CrLPAAT1_; see Figure 1a) was amplified by PCR for TOPO cloning (pENTR/D‐TOPO vector, Invitrogen, Carlsbad, CA), using the primers CrLPAAT1FW+CACC (5′‐CACCATGGCAAGGAAGTCTTCGCTGG‐3′)/CrLPAAT1TPRV (5′‐GATTTTTGCCAGAAGAACATTCGG‐3′). *TP*_*CrLPAAT1*_ was then subcloned into pGWB505, so that TP_CrLPAAT1_ was fused in‐frame to the N‐terminus of the superfolder green fluorescent protein (sGFP) and expression was driven by the cauliflower mosaic virus 35S promoter (Nakagawa *et al*., 2007). The resultant construct was infiltrated into *Nicotiana benthamiana* leaves as described previously (Sparkes *et al*., 2006). sGFP fluorescence was observed using a confocal laser scanning microscope (Olympus) by excitation with a 488‐nm laser and detection between 500 and 530 nm. Chlorophyll fluorescence was observed independently with excitation at 488 nm and emission from 650 to 700 nm.

### Subcellular localization of CrLPAAT1 in *Chlamydomonas*

Genomic DNA of *CrLPAAT1* was amplified using the primers CrLPAAT1‐F (5′‐CATATGGCGCGTAAAAGCAGTTTGGC‐3′)/CrLPAAT1‐R (5′‐AGATCTCTGCTCATCCGGCGCCATCTCT‐3′). The resultant *CrLPAAT1* fragment was subcloned between the *Nde*I and *Bgl*II sites of pOpt‐Clover‐Paro (Lauersen *et al*., 2015) to create pOpt‐CrLPAAT1‐Clover‐Paro. The *Chlamydomonas* wild‐type strain CC‐125 was then transformed with pOpt‐CrLPAAT1‐Clover‐Paro by electroporation (Shimogawara *et al*., 1998). Cells were then excited with a 488‐nm laser, and Clover fluorescence was collected between 500 and 530 nm, and chlorophyll fluorescence was collected between 650 and 700 nm.

### Overexpression of CrLPAAT1 in the plastid genome: vector construction, plastid transformation, genotyping and immunoblot

Codon‐optimized *CrLPAAT1* for expression in *C. reinhardtii* plastids (Kazusa Codon Usage Database at: http://www.kazusa.or.jp/codon/) was synthesized by GenArts (Invitrogen). The synthetic gene (designated *CrLPAAT1crc*) was then amplified using the primers InFLPAAT‐F (5′‐ AAATCCATGGAGATCTTAATGGCTCGTAAATCATCATTAG‐3′)/InFLPAAT‐R (5′‐ACGTTTAAACAGATCTTTATGATGAAGCAGCATAATC‐3′), and the resultant fragment was subcloned into the plastid expression vector pLM21 as described (Michelet *et al*., 2011). To enable screening for transformants by immunoblot, *CrLPAAT1crc* was expressed as a C‐terminal fusion to the triple influenza hemagglutinin epitope (3xHA) (the construct was named *pLM21‐CrLPAAT1crc‐HA*). The HA epitope has been successfully used to determine the subcellular localization of several proteins in *C. reinhardtii,* including the plastid‐located ω‐3 fatty acid desaturase 7 (Nguyen *et al*., 2013).

For plastid transformation, the plasmid DNA (*pLM21‐CrLPAAT1crc‐HA*) was precipitated on nanometre‐scale gold particles using the Seashell Technology S550d gold DNA protocol (Seashell Technology, La Jolla, CA). Exponentially growing cells were harvested and spread out evenly on a TAP agar plate containing spectinomycin (100 μg/mL). The cells on the plates were then bombarded with plasmid‐coated gold particles at high pressure (7 kg/cm^2^) using a helium gun (Michelet *et al*., 2011). After transformation, the plates were left in the dark for up to 24 h to let the cells recover. Antibiotic‐resistant clones started to appear after around 10 days.

As a single plastid in *C. reinhardtii* contains 50–80 copies of the circular plastidial genome (Day and Goldschmidt‐Clermont, 2011), before chemical phenotypic analyses, antibiotic‐resistant clones were first genotyped for the correct integration of the full‐length *CrLPAAT1* gene into the plastid genome using gene‐specific primers (InFLPAAT‐F and InFLPAAT‐R). The state of homoplasmy was then verified using the primers IRR pLM21.2 (5′‐CGTTATTAGCCTTTCGTCGCT‐3′)/pLM20*Cla*I (5′‐CGAAACGGTGGTTATTCCAGGCC‐3′). For this, a rapid total DNA extraction was performed using Chelex 100 (Sigma) (Werner and Mergenhagen, 1998). The integrity and presence of plastid DNA was tested by amplification of the plastidial 16S ribosomal DNA using the primers 16S‐23F (5′‐TCCATGGAGAGTTTGATCCTG‐3′)/16S‐300R (5′‐TCCTCTCAGACCAGCTACTGC‐3′). GoTaq Polymerase (Promega, Madison) was used for PCR amplification.

Wild type and plastid transformants of *CrLPAAT1* were grown to the exponential phase and 10 million cells were harvested for protein extraction and immunoblot analysis following a previously described method (Nguyen *et al*., 2013). Briefly, around 20 μg of protein was loaded onto a 12% Bis Tris SDS‐PAGE gel (Thermo Fisher Scientific, Waltham, MA). After migration, proteins were transferred to nitrocellulose membrane using a semidry transfer technique. Immunodetection was performed after overnight hybridization with anti‐HA rat antibodies (Roche, Basel, Switzerland) followed by incubation with a secondary antibody (anti‐rat IgG peroxidase, Sigma) for 45 min. Immobilon^™^ Western Chemiluminescent HRP (horseradish peroxidase) Substrate (Merck millipore, Billerica, MA) was used for the detection and images were recorded using a G:BOX Chemi XL (Syngene, Cambridge, UK).

### RNA extraction and quantitative real‐time RT‐PCR (qPCR)

Total RNA was extracted according to a phenol/chloroform method described previously (Kim *et al*., 2013), and cDNA was synthesized by reverse transcription using 2 mg of total RNA. Real‐time PCR was performed using primer sets designed to amplify *CrLPAAT1* and *RACK1* using the primer pairs CrLPAAT1‐qPCR‐F (5′‐ CAACTCAGACTCCGCTCCTCAG‐3′)/CrLPAAT1‐qPCR‐R (5′‐ GTACTTGTCGAACGCCAGCACG‐3′) and RACK1‐F (5′‐ GACCACCAACCCCATCAT‐3′)/RACK1‐R (5′‐ AGACGGTCACGGTGTTGA‐3′), respectively.

### Lipid extraction and lipid molecular species analysis via liquid chromatography–tandem mass spectrometry (LC‐MS/MS)

Cells at the exponential phase were harvested by centrifugation at 600 ***g*** for 4 min at 4 °C. The lipid extraction method and LC‐MS/MS settings were described in (Legeret *et al*., 2015). Briefly, cell pellets (10 million cells) were quenched immediately by adding 1 mL of preheated isopropanol (at 85 °C) containing butylated hydroxytoluene (0.01%, v/v), to which 1 μg each of the internal standards phosphatidylethanolamine (PtdEtn17:0/17:0) and triacylglycerol (TAG17:0/17:0/17:0) were added. The mixtures were vortexed and heated up (at 85 °C) for an additional 10 min to deactivate lipases. After cooling, methyl tert‐butyl ether (MTBE, 3 mL) was used to extract the lipids. MTBE extraction was repeated a second time, and the extracted lipids were then dried under a flow of nitrogen gas, dissolved in a solvent mixture (acetonitrile:isopropanol:10 mm ammonium acetate = 65 : 30 : 5, by volume) and analysed by LC‐MS/MS.

## Conflict of interest

The authors declare there is no conflict of interests.

## Supporting information


**Figure S1** Confirmation of homoplasmy for *CrLPAAT1* expression in the plastid genome.
***Figure S2** Membrane lipid molecular species of the LPAAT overexpressors cultivated in TAP medium lacking nitrogen (TAP‐N).*
**Figure S3** Membrane lipid molecular species of the LPAAT overexpressors cultivated in normal TAP medium.
