# Supplementary material for: Identification of a Chlamydomonas plastidial 2‐lysophosphatidic acid acyltransferase and its use to engineer microalgae with increased oil content
Source: Plant Biotechnol J. 2016 May 23;14(11):2158–67. doi: 10.1111/pbi.12572 (PMC5096022; doi:10.1111/pbi.12572)
Supplement: Supplementary file 1 — Figure S1 Confirmation of homoplasmy for CrLPAAT1 expression in the plastid genome. Figure S2 Membrane lipid molecular species of the LPAAT overexpressors cultivated in TAP medium lacking nitrogen (TAP‐N). Figure S3 Membrane lipid molecular species of the LPAAT overexpressors cultivated in normal TAP medium. [file PBI-14-2158-s001.docx]

**Supplemental material**


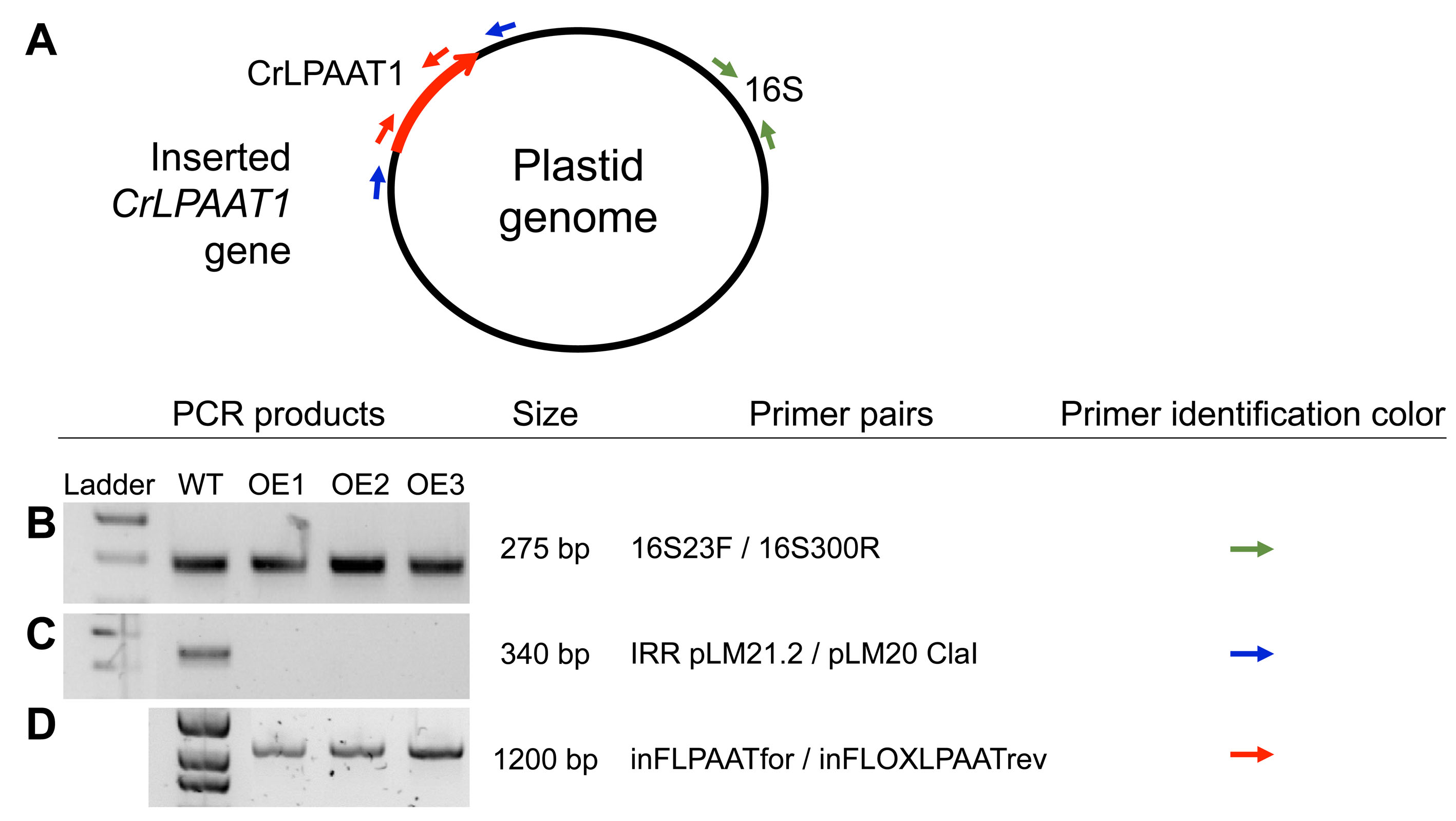


**Supplemental Figure S1. Confirmation of homoplasmy for *CrLPAAT1* expression in the plastid genome.**

(**A**) A schematic diagram showing the integration of *CrLPAAT1* (large red arrow) into the plastid genome of *C. reinhardtii*. Small arrow pairs correspond to primers in B-D.

(**B**) Verification of proper extraction of the plastidial DNA.

(**C**) Confirmation of the integration events as demonstrated by the absence of the PCR product around the integration site in the plastid genome.

(**D**) Confirmation of the presence of the inserted codon-optimized *CrLPAAT1* gene.


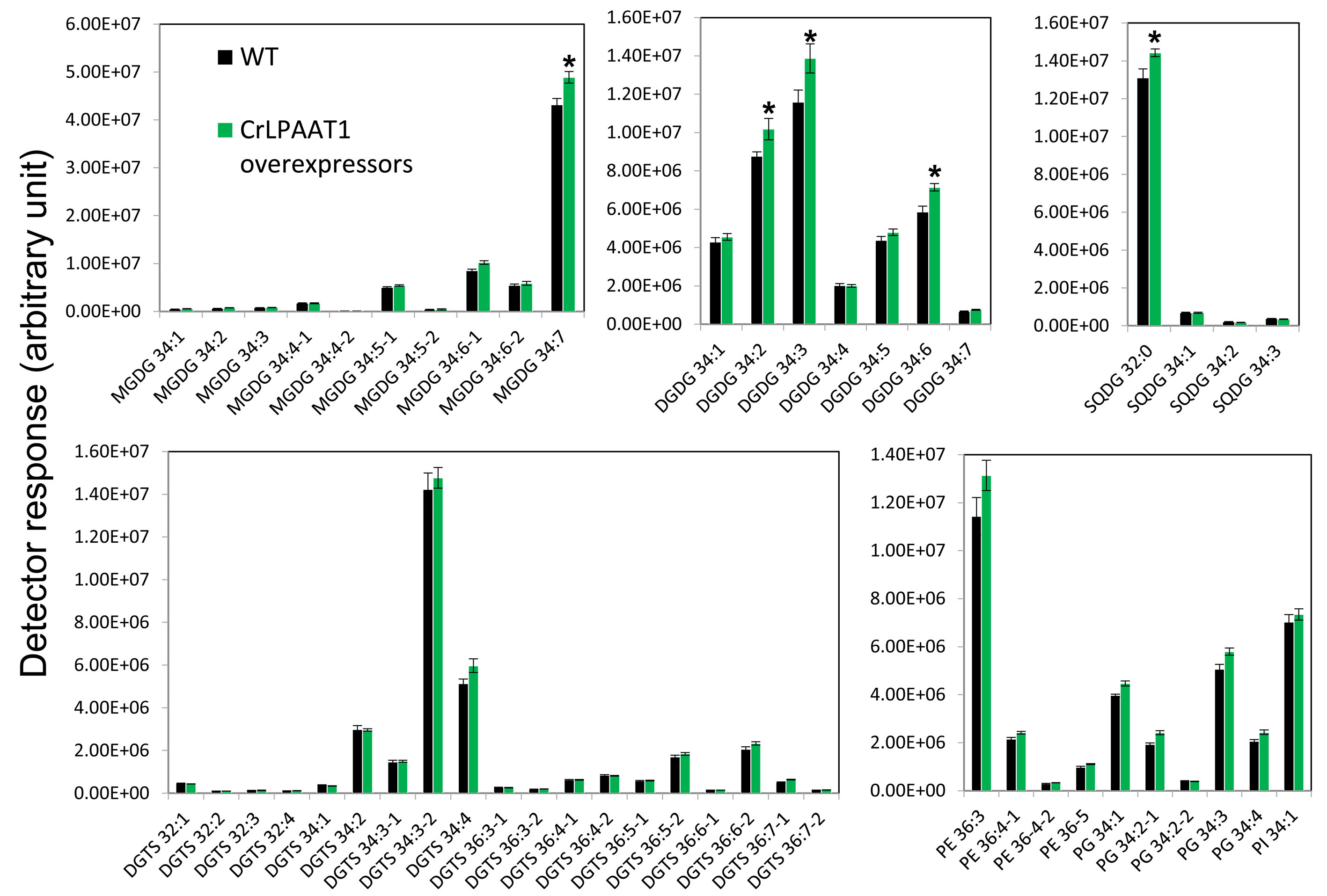


**Supplemental Figure S2. Membrane lipid molecular species of the LPAAT overexpressors cultivated in TAP medium lacking nitrogen (TAP-N).**

Data are means of three biological replicates (OE1, OE2, and OE3 shown in Figure 6) together with three technical replicates for each biological replicate; error bars denote 95% confidence intervals. *: indicates significant changes. Statistical analysis was carried out using the Student's *t*-test (*p*<0.05). Black bars, wild type; gray bars, CrLPAAT1 overexpressors. Lipid molecular species are noted as the name of the lipid class (total number of carbons: total number of unsaturation); numbers following a dash denote the occurrence of isomers for that particular lipid species.


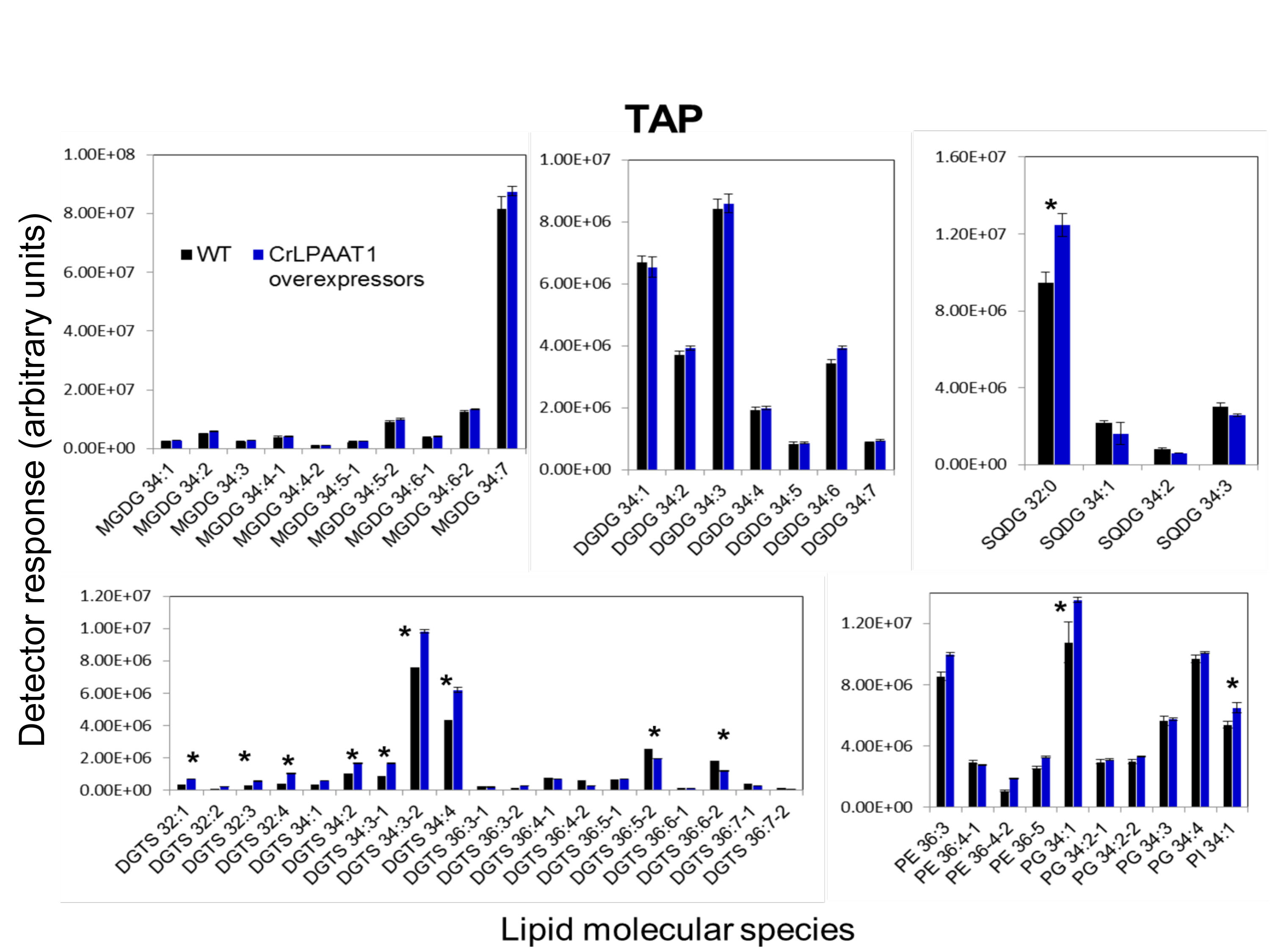


**Supplemental Figure S3. Membrane lipid molecular species of the LPAAT overexpressors cultivated in normal TAP medium.**

Data are means of three biological replicates (OE1, OE2, and OE3 shown in Figure 6 before nitrogen starvation) together with three technical replicates for each biological replicate; error bars denote 95% confidence intervals. * indicates significant changes. Statistical analysis was carried out using Student's *t*-test (p<0.05). Black bars, wild type; blue bars, CrLPAAT1 overexpressors. Lipid molecular species are noted as the name of lipid class (total number of carbons: total number of unsaturation); numbers following a dash denote the occurrence of isomers for that particular lipid species.
